# Psychological impact of the state of emergency over COVID-19 for non-permanent workers: a Nationwide follow-up study in Japan

**DOI:** 10.1186/s12889-021-10401-y

**Published:** 2021-02-11

**Authors:** Shota Saito, Huyen Thi Thanh Tran, Ruan Qi, Kenji Suzuki, Toru Takiguchi, Kazuo Ishigami, Shinichi Noto, Sachiko Ohde, Osamu Takahashi

**Affiliations:** 1 Center for Clinical Epidemiology and Health Technology Assessment, St. Luke’s International University, OMURA Susumu & Mieko Memorial St. Luke’s Center for Clinical Academia, 5th Floor 3-6-2 Tsukiji Chuo-ku, Tokyo, 104-0045 Japan; 2 Field of Health Informatics and Business Administration, Graduate School, Niigata University of Health and Welfare, Niigata, Japan; 3 Department of Occupational Therapy, Niigata University of Health and Welfare, Niigata, Japan; 4 Graduate School of Public Health, St. Luke’s International University, Tokyo, Japan; 5 Department of General Internal Medicine, St. Luke’s International Hospital, Tokyo, Japan

**Keywords:** COVID-19, General workers, Mental health, Propensity score analysis, Quality of life, Employment security, Unemployment, Web survey

## Abstract

**Background:**

The outbreak of COVID-19 has caused mental health problems and increased unemployment due to the economic recession. This survey aimed to assess the psychological impact of the state of emergency. We estimated changes in mental health, quality of life, and unemployment experience for general workers during the first COVID-19 outbreak in Japan.

**Methods:**

We conducted a nationwide follow-up study. During the periods of March 26 to April 6, 2020 and June 26 to July 2, 2020, we used the internet to survey general workers aged 15 to 59 years in Japan. The questionnaire items covered employment status and socioeconomic factors, and we used the Center for Epidemiologic Studies Depression Scale (CES-D) and EQ-5D-5L to assess depression and health-related quality of life (HR-QOL), respectively. The differences in outcomes of permanent and non-permanent workers were analyzed using propensity score analysis. A multiple linear regression analysis was performed to examine the relationship between unemployment and CES-D scores.

**Results:**

We included 2351 subjects in the analysis. Changes in both CES-D scores and utility were not significantly different between the two groups. However, a significant difference was found regarding the rate of unemployment, which was associated with higher CES-D scores.

**Conclusions:**

The present study demonstrated that the mental health of non-permanent workers was not negatively affected following the state of emergency due to COVID-19 in Japan. Unemployment is an important factor that influences the mental health of general workers.

## Background

The coronavirus disease has significantly impacted global public health and has been spreading worldwide. As of August 3, 2020, 17,918,582 COVID-19 cases have been confirmed and 686,703 people have died of the disease worldwide [1]. Many countries have closed their external borders and imposed nationwide lockdowns to successfully—though temporarily—control the spread of COVID-19. However, the countermeasures have led to reduced workforces across all economic sectors, resulting in many job losses [2].

In Japan, the government declared a state of emergency in seven prefectures, including Tokyo and Osaka, on April 7, 2020. Following an upsurge in the “untraceable” new infections, the state of emergency was expanded to the whole of Japan, and 13 prefectures were designated as “special alert areas” on April 16. With the effectuation of the state of emergency, the government urged citizens to refrain from going outside, practice social distancing, stay at home, and follow travel restrictions; non-essential businesses were closed for about a month [3, 4].

The outbreak of COVID-19 has caused mental health problems and increased unemployment due to the economic recession. A large-scale cross-sectional study in China indicated that being at work was associated with lower risks of depression, anxiety, and insomnia [5]. Several studies reported that the rates of depression and anxiety were 18.7 and 21.6%, respectively, among Spanish people during the initial stage of the COVID-19 outbreak; Spanish adults confined due to COVID-19 restrictions of movement showed inverse associations between current physical activity and current perceived anxiety and mood [6, 7].. The Organization for Economic Co-operation and Development (OECD) indicated that the unemployment rate increased 2.9%, reflecting the impact of COVID-19 containment measures [2]. Recently, the Japanese government reported that the mental health conditions of unemployed and non-permanent workers may be particularly vulnerable during the ongoing COVID-19 crisis [8].

The number of non-permanent workers has been increasing in Japan. Precarious employment includes part-time, dispatched, and fixed-term work, and these accounted for 22 and 53% of all paid employment for males and females, respectively, in 2020 [9]. There was an adverse effect on the mental health of non-permanent workers due to employment instability, and unemployment status was related to psychological states such as depression, anxiety, and poor health outcomes due to job loss.

A previous study suggested that precarious employment is associated with double the risk of serious psychological distress among middle-aged Japanese men [10]. Moreover, the transition from full-time permanent employment to another employment status was also associated with the onset of severe depressive symptoms in East Asia [11, 12]. It is well known that poor mental health status, including depression, is an independent risk factor for suicide and is associated with a lower quality of life compared to healthy subjects [13, 14].

The current focus on the COVID-19 outbreak is not only the medical outcomes of infected patients but also the mental health of affected individuals and the general population. Therefore, it is meaningful to investigate the changes in mental health and quality of life during the COVID-19 crisis. No study has yet examined the association between employment security and mental health problems after the lifting of the state of emergency in Japan. The aim of this study is to assess the psychological impact of the state of emergency. We estimated changes in mental health, quality of life, and unemployment experience for general workers during the first COVID-19 outbreak in Japan.

## Methods

### Study design and data collection

This nationwide survey was administered online to general workers in Japan aged 15 to 59 years through a platform of more than two million candidates. It was administered by the Cross Marketing Corporation, Tokyo, Japan, which specializes in questionnaire research.

**Fig. 1:**
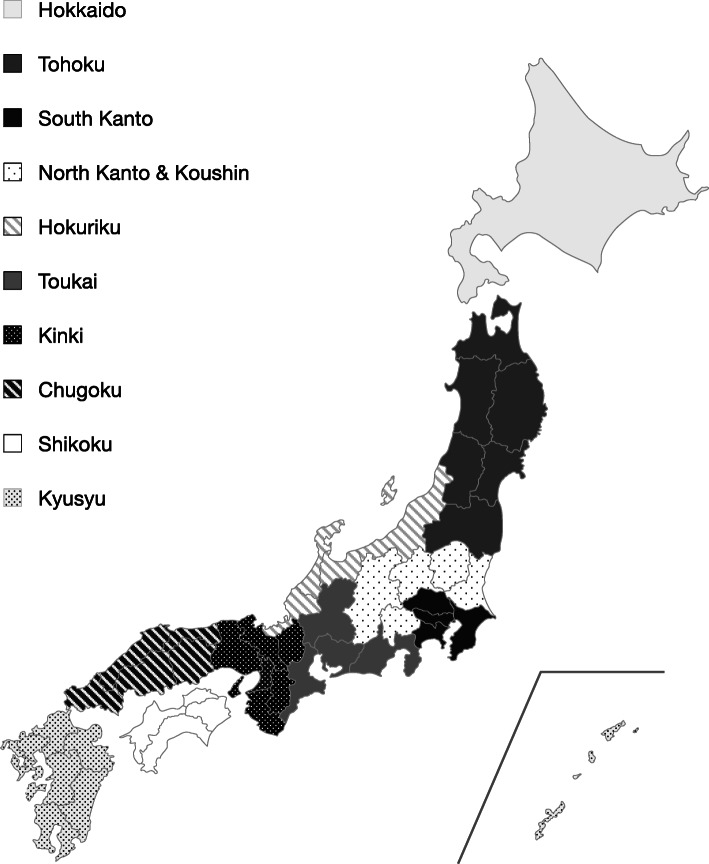
The ten geographical regions of Japan

The initial survey was conducted from March 26 to April 6, 2020, before the state of emergency for COVID-19 was declared. The follow-up survey was administered to the same cohort of respondants after the state of emergency was lifted, between June 26 and July 2, 2020. The initial survey was administered until data had been collected from 3000 respondents, and the response rate for the follow-up survey targeted 70% of the initial cohort on the basis of the general response rate for web-based surveys. In the initial survey, we ensured a representative sample of the Japanese population with regards to age, sex, and residential area. The residential area in Japan was divided into 10 regions (Fig. 1). We did not provide any incentives or compensation to the participants for this study.

### Questionnaire

#### Working status

We defined working status as the four types mentioned below; the subjects selected a status during their self-reports.

Permanent employees: Company employees who are guaranteed lifetime employment until retirement, are hired directly by their employers, and have full-time employment.

Non-permanent employees: Company employees with fixed-term labor contracts such as part-time employees, dispatched employees from temporary labor agencies, and contract or entrusted employees.

Civil servant: Public service workers in national or local governments who are incorporated into nonprofit organizations.

Self-employed: Self-employed individuals such as sole proprietors and freelancers.

The target population of this study was workers employed by general companies. We excluded civil servants because in Japan, they are guaranteed employment until retirement. We also excluded self-employed individuals because they are not employees.

#### Socioeconomic status and medical conditions

The questionnaires covered age, sex, region, marital status, children, family members living together, education, industry, company size, personal income, family income, average overtime per month, labor union membership, householder, exercise, smoking history, drinking history, commute time, and average sleeping time.

We also collected information regarding past medical history such as heart disease, cerebrovascular disease, cancer, Alzheimer’s disease, physical disorders with chronic pain, epilepsy, and depression, which are associated with mental conditions, according to the Diagnostic and Statistical Manual of Mental Disorders – Fifth Edition (DSM-5) [15]. The number of diseases was defined as a primary factor in this analysis. We used the Japanese version of the sense of coherence (SOC) scale to measure stress-coping ability, with a final score ranging from 13 to 91. This scale included 13 items, with higher scores indicating better stress-coping ability [16].

#### Outcomes

In both the first and second surveys, the degree of depressive symptoms and HR-QOL were assessed. We used the Japanese version of the CES-D to measure depressive symptoms. This scale consisted of 20 items asking participants to rate how often they had experienced symptoms associated with depression over the past week. CES-D scores ranged from 0 to 60, with scores above 16 usually indicating depressive symptoms. The CES-D has good sensitivity, specificity, and high internal consistency for identifying the risk of depression [17].

We used the five-dimension EQ-5D-5L instrument to assess the respondents’ HR-QOL. The EQ-5D-5L consists of five items—namely, mobility, self-care, usual activities, pain/discomfort, and anxiety/depression—rated across five levels. The resulting generic preference-based measure reflects subjective values assigned to specific health-related outcomes ranging from − 0.025 to 1, with 0 indicating death and 1 indicating perfect health in the Japanese value set. We call this score the utility weight [18].

The differences in CES-D scores and utility values between the two timeframes were defined as outcomes in this analysis. We also investigated the unemployment experience during the emergency period in the second survey.

### Statistical analysis

Propensity score matching was performed to adjust for confounding factors between the permanent and non-permanent groups and to evaluate outcomes [19]. Propensity scores were estimated using a multiple logistic regression model with socioeconomic factors and clinical indicators such as comorbidity and baseline CES-D score and utility. According to their propensity scores, we conducted nearest-neighbor matching with a caliper of 0.2 standard deviations of the propencity score at a 1:1 ratio, without replacement, using the estimated propensity score. In the matched subjects, absolute standardized differences in the means and proportions of those variables were used to confirm the propensity scoring balance between the two groups.

In the matched cohort, we compared the CES-D score changes, utility change, and rate of unemployment experience between permanent and non-permanent employees. Pearson’s chi-squared test was used to compare categorical variables, and a Student’s *t*-test was used to compare continuous variables. Finally, we assessed the psychological impact of unemployment in terms of changes in CES-D scores. A multiple linear regression analysis was performed to identify the determinants of the changes. Independent variables used in the analysis were working status, unemployment experience, sex, age, number of comorbidities, region, marital status, personal income, family income, average work time per day, labor union membership, householder, exercise, smoking, drinking, average sleeping time, SOC score, and baseline CES-D score. Categorical variables and ordered variables were converted into dummy variables. We considered the interaction of working status and unemployment experience to assess the degree of psychological impact in the two groups.

All statistical tests were two-sided, and *p*-values less than 0.05 were considered significant. All analyses were conducted using STATA 16.1 (College Station, Texas, USA: StataCorp LP).

## Results

### Data collection

**Fig. 2:**
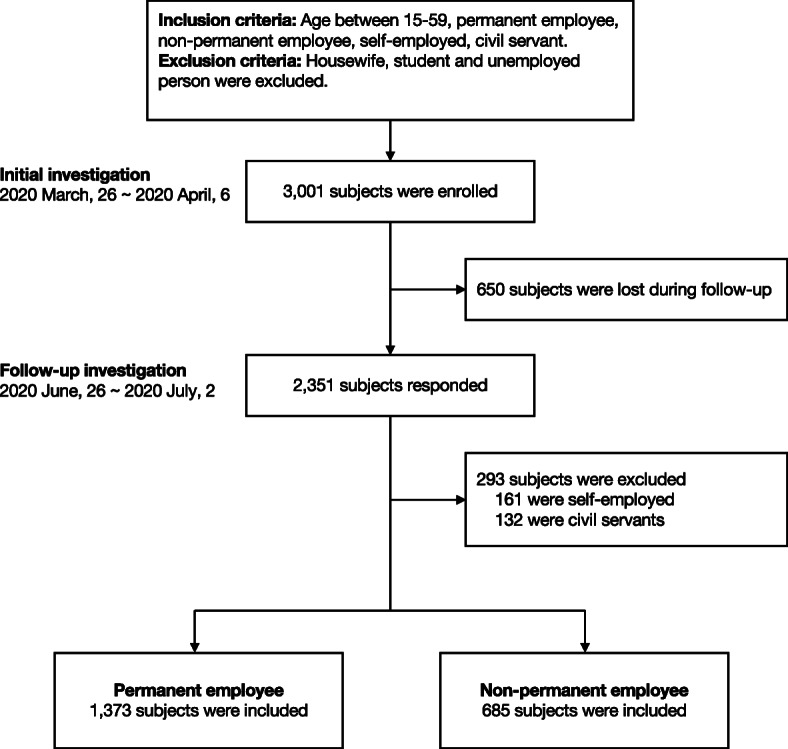
Flowchart of the participants in the study

We collected data from 3001 subjects (excluding housewives, students, and unemployed people) in the first survey, and 2351 subjects responded to the follow-up survey. We then excluded 161 self-employed individuals and 132 civil servants from the analysis. Finally, 1373 permanent employees and 685 non-permanent employees were included in the propensity score matching as the permanent and non-permanent groups, respectively (Fig. 2).

### Propensity score analysis

Propensity score matching identified 497 subjects from both the non-permanent and permanent groups. Therefore, we included a total of 994 subjects in the subsequent analyses. Table 1 shows the differences between the baseline characteristics of the non-permanent and permanent groups before and after the matching. All baseline variables included in the model were well-balanced within the standardized difference or close to 0.1 after matching. The c-statistic of the propensity score was estimated to be 0.862, ranging from 0.846 to 0.878, indicating good discrimination between the two groups. We were able to collect the data without any missing values using the web-based survey.

**Table 1:** Baseline characteristics before and after propensity score matching

		Before matching (*n* = 2648)					After matching (*n* = 994)				
Variable	Category	Permanent employee(*n* = 1373)	%	Non-permanent employee(*n* = 685)	%	Standardized difference	Permanent employee(*n* = 497)	%	Non-permanent employee(n = 497)	%	Standardized difference
Age	Mean (SD)	40.78(10.54)		40.22(11.64)		0.0501	39.04(10.96)		39.64(11.47)		−0.0536
Sex	Male	820	59.7	202	29.5	0.6385	176	35.4	175	35.2	0.0042
	Female	553	40.3	483	70.5		321	64.6	322	64.8	
Region^a^	Low infection area	904	65.8	433	63.2	0.0550	349	70.2	337	67.8	0.0522
	High infection area	469	34.2	252	36.8		148	29.8	160	35.2	
Marital status	Single	674	49.1	356	52.0	0.0577	294	59.2	277	55.7	0.0692
	Married	699	50.9	329	48.0		203	40.8	220	44.3	
Child	No	846	61.6	439	64.1	0.0512	346	69.6	330	66.4	0.0691
	Yes	527	38.4	246	35.9		151	30.4	167	33.6	
Family members living together	0	169	12.3	59	8.6	0.1673	59	11.9	51	10.3	0.0938
	1	355	25.9	152	22.2		124	24.9	112	22.5	
	2	332	24.2	177	25.8		131	26.4	132	26.6	
	3	287	20.9	165	24.1		99	19.9	107	21.5	
	4 ≤	230	16.8	132	19.3		84	16.9	95	19.1	
Comorbidities	0	1250	91.0	606	88.5	0.0854	447	89.9	441	88.7	0.0399
	1	110	8.0	70	10.2		45	9.1	50	10.1	
	2 ≤	13	0.9	9	1.3		5	1.0	6	1.2	
Education	Jr high school	13	0.9	35	5.1	0.6319	9	1.8	13	2.6	0.0957
	High school	309	22.5	246	35.9		157	31.6	168	33.8	
	Associate degree or Diploma	293	21.3	219	32.0		159	32.0	151	30.4	
	Bachelor	652	47.5	169	24.7		160	32.2	149	30.0	
	Master or Doctor	106	7.7	16	2.3		12	2.4	16	3.2	
Industry^d^	Primary sector	10	0.7	4	0.6	0.3550	6	1.2	4	0.8	0.0727
	Secondary sector	422	30.7	112	16.4		107	21.5	97	19.5	
	Tertiary sector	891	64.9	526	76.8		362	72.8	370	74.4	
	Industries unable to classify	50	3.6	43	6.3		22	4.4	26	5.2	
Company size (number of employees)	< 50	410	29.9	287	41.9	0.3959	214	43.1	210	42.3	0.0340
	50 ~ 99	158	11.5	128	18.7		75	15.1	75	15.1	
	100 ~ 299	209	15.2	71	10.4		59	11.9	61	12.3	
	300 ~ 999	221	16.1	79	11.5		65	13.1	62	12.5	
	1000 ≤	375	27.3	120	17.5		84	16.9	89	17.9	
Personal income^b^	Low	623	45.4	653	95.3	1.3067	468	94.2	465	93.6	0.0466
	Middle	549	40.0	22	3.2		2	4.4	22	4.4	
	High	201	14.6	10	1.5		7	1.4	10	2.0	
Family income^b^	Low	323	23.5	305	44.5	0.5459	237	47.7	227	35.7	0.0405
	Middle	562	40.9	270	39.4		174	35.0	180	36.2	
	High	488	35.5	110	16.1		86	17.3	90	18.1	
Average overtime per month	< 10 h	715	52.1	581	84.8	0.7640	384	77.3	396	79.7	0.1014
	10 ~ 44 h	502	36.6	91	13.3		91	18.3	88	17.7	
	45 ~ 79 h	127	9.2	10	1.5		17	3.4	10	2.0	
	80 h ≤	29	2.1	3	0.4		5	1.0	3	0.6	
Union membership	No	940	68.5	546	79.7	0.2588	384	77.3	390	78.5	0.0291
	Yes	433	31.5	139	20.3		113	22.7	107	21.5	
Householder	No	481	35.0	481	70.2	0.7530	289	58.1	315	63.4	0.1073
	Yes	892	65.0	204	29.8		208	41.9	182	36.6	
Exercise^c^	No/custom	799	58.2	470	86.8	0.2241	328	66.0	329	66.2	0.0475
	Once every 2 weeks	140	10.2	47	6.9		42	8.5	36	7.2	
	Once in a week	197	14.3	70	10.2		54	10.9	56	11.3	
	Two times in a week	237	17.3	98	14.3		73	14.7	76	15.3	
Smoking	No	909	66.2	520	75.9	0.2188	372	74.8	370	74.4	0.0142
	Yes	295	21.5	99	14.5		81	16.3	81	16.3	
	Past	169	12.3	66	9.6		44	8.9	46	9.3	
Drink	No	588	42.8	362	52.8	0.2259	243	48.9	254	51.1	0.0443
	Yes	717	52.2	281	41.0		230	46.3	220	44.3	
	Past	68	5.0	42	6.1		24	4.8	23	4.6	
Commute time (one-way)	< 30 min	686	50.0	433	63.2	0.3038	300	60.4	302	60.8	0.0419
	30 ~ 59 min	475	34.6	198	28.9		146	29.4	150	30.2	
	60 min ≤	212	15.4	54	7.9		51	10.3	45	9.1	
Sleeping time	< 4.0 h	35	2.5	15	2.2	0.1023	12	2.4	12	2.4	0.0504
	4.0–5.9 h	351	25.6	148	21.6		122	24.5	112	22.5	
	6.0 ~ 7.9 h	843	61.4	440	64.2		305	61.4	316	63.6	
	8.0 h ≤	144	10.5	82	12.0		58	11.7	57	11.5	
SOC score	Mean (SD)	52.90(9.77)		52.50(10.74)		0.0384	52.11(10.20)		51.99(10.32)		0.0218
Base-line CES-D score	Mean (SD)	18.04(10.62)		18.76(10.72)		−0.0675	19.19(10.94)		18,95(10.81)		0.0222
Base-line utility	Mean (SD)	0.8921(0.15723)		0.8853(0.13672)		0.0461	0.8867(0.15697)		0.8854(0.13332)		0.0092

CES-D; Center for Epidemiologic Studies Depression Scale, SOC; Sense of coherence, SD; Standard deviation

^a^ High infection area includes Tokyo, Saitama, Chiba, Kanagawa, and Hokkaido.^b^ Low (less then JPY 4 million), Middle (between JPY 4 million to JPY 8 million), High (more than JPY 8 million)^c^ Exercise is defined as moderate exercise with light-breathing for about 1 h^d^ The industries were categorized as four types: the primary sector including agriculture and forestry, fisheries; the secondary sector including mining and quarrying of stone and gravel, construction, manufacturing; the tertiary sector including electricity gas heat supply and water, information and communications, transport and postal activities, wholesale trade and retail trade, finance and insurance, real estate and goods rental and leasing, scientific research, professional and technical services, accommodations, eating and drinking services, living-related and personal services and amusement services, education learning support, medical health care and welfare, compound services, other services, government; and industry unable to classify

**Fig. 3:**
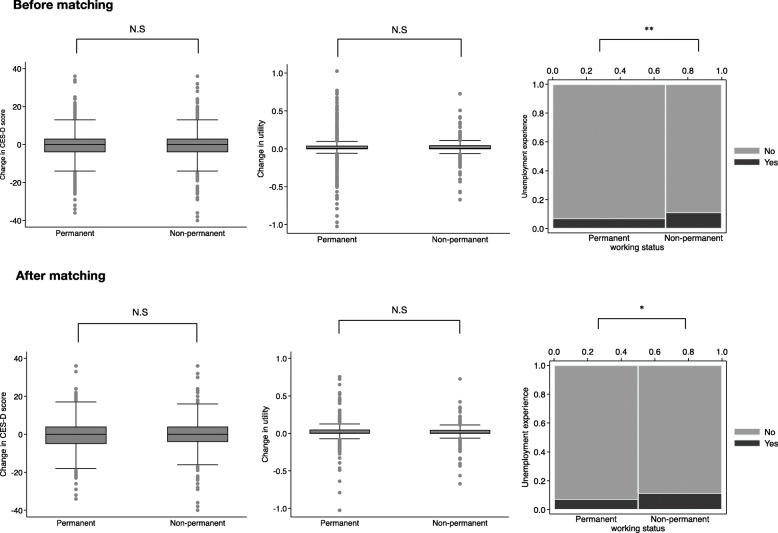
Box plot of changes in CES-D score and health-related utility, and spine plot of unemployment experience between permanent and non-permanent groups. (N.S: Not significant, *: *p* < 0.05, **: *p* < 0.01, ***: *p* < 0.001)

A comparison of changes in the CES-D scores, utility changes, and rates of unemployment between the two groups is shown in Fig. 3. The changes in CES-D scores were estimated to be − 0.706 for permanent employees and − 0.575 for non-permanent employees *(p* = 0.807). The utility change also did not differ significantly between the two groups (permanent 0.014 vs. non-permanent 0.009, *p* = 0.533). However, there was a significant difference regarding rate of unemployment, and the data matched (permanent 7.20% vs. non-permanent 11.47%, *p* = 0.022); the risk ratio of unemployment was estimated to be 1.583 (95% confidence interval = 1.063–2.358).

### Multiple linear regression analysis

The results of the multiple linear regression analysis as presented in Table 2 showed that the unemployment experience was a factor associated with increasing the CES-D score (*p* = 0.003). There were no significant differences in working status and its interaction with unemployment (*p* = 0.340). More than two comorbidities (*p* = 0.044) and average work time per day between 10 to 12 h (*p* = 0.027) were associated with higher CES-D scores. Specifically, a high SOC score (*p* < 0.001), married status (*p* = 0.032), and a baseline CES-D score (*p* < 0.001) were associated with lower CES-D scores.

**Table 2:** Multiple linear regression models of socioeconomic indicators and changes in the CES-D score (*N* = 2351)

Variables	Coefficient	Lower 95% CI	Upper 95% CI	*P* value	
Non-permanent	0.135	−0.720	0.989	0.757	
Unemployment experience	2.358	0.793	3.923	0.003	**
Non-permanent * Unemployment experience	−1.148	−3.507	1.210	0.340	
Sex	−0.301	−1.055	0.452	0.433	
Age	−0.015	−0.050	0.020	0.397	
Number of comorbidities					
0 (base)	1.000				
1	0.939	−0.238	2.116	0.118	
More than 2	3.284	0.092	6.476	0.044	*
High infection area	0.566	−0.125	1.256	0.108	
Married	−0.821	−1.570	−0.072	0.032	*
Personal income					
Low (base)	1.000				
Middle	−0.078	−1.122	0.967	0.884	
High	−0.551	−2.099	0.998	0.486	
Family income					
Low (base)	1.000				
Middle	−0.465	−1.417	0.486	0.338	
High	−1.003	−2.189	0.184	0.098	
Average work time per day					
Less than 8 h (base)	1.000				
Between 8 and 10 h	−0.091	− 0.888	0.706	0.822	
Between 10 and 12 h	1.574	0.179	2.969	0.027	*
Over 12 h	0.467	−2.210	3.145	0.732	
Union membership	−0.194	−0.928	0.540	0.604	
Householder	0.235	−0.617	1.087	0.589	
Exercise					
None (base)	1.000				
Once every 2 weeks	0.531	−0.639	1.701	0.373	
Once in a week	0.537	−0.472	1.545	0.297	
More than 2 times in a week	−0.203	−1.126	0.719	0.665	
Smoking					
No (base)	1.000				
Yes	−0.270	−1.151	0.611	0.548	
Past	−0.338	−1.441	0.766	0.548	
Drink					
No (base)	1.000				
Yes	−0.604	−1.306	0.097	0.091	
Past	0.011	−1.527	1.549	0.989	
Sleeping time					
Less than 4 h	0.750	−1.381	2.880	0.490	
Between 4 and 6 h	−0.052	− 0.838	0.735	0.898	
Between 6 and 8 h (base)	1.000				
Over 8 h	0.999	− 0.074	2.073	0.068	
SOC score	−0.134	−0.172	− 0.097	< 0.001	***
Base-line CES-D score	−0.383	−0.420	− 0.347	< 0.001	***
Constant	14.721	11.913	17.528	< 0.001	***

*CES-D* Center for Epidemiologic Studies Depression Scale, *SOC* Sense of coherence

*: P < 0.05, **: P < 0.01, ***: P < 0.001

## Discussion

The physiological impact of the COVID-19 outbreak is a global concern. In the present study, we examined changes in CES-D scores, health-related utility, and unemployment of general workers during the COVID-19 state of emergency based on data from a Japanese nationwide web-based questionnaire. There were no statistically significant differences in these scores after matching the backgrounds of the subjects. However, unemployment in the non-permanent group was statistically higher than in the permanent group, even after adjusting for baseline factors. Our findings suggest that there was a deterioration of employment conditions, especially for non-permanent workers, after the state of emergency. Unemployment was found to worsen the psychological conditions of general workers in Japan.

Several studies have demonstrated the predictive factors associated with increasing depression and anxiety. In Japan, a cohort study conducted by Sairenchi et al. [20] revealed that SOC may be able to predict the onset of depression in Japanese workers, and Urakawa et al. [21] reported that increasing SOC may reduce negative job stress responses and subjective symptoms for general workers. Recently, a large-scale cross-sectional study conducted by Kikuchi et al. [22] clarified that Japanese workers with longer overtime showed significantly higher anxiety and depression than those with less overtime, among both males and females. Moreover, a Korean study suggested that head of household status, sex, and precarious employment were associated with the onset of severe depressive symptoms [11].

In the propensity score analysis, we adjusted those factors between the permanent and non-permanent groups. There was no statistically significant difference in CES-D scores between the groups; however, there was a slight improvement in the scores of both groups. Although non-permanent workers reported higher rates of unemployment compared to workers with permanent contracts, a significant impact on mental health was not observed in our data. Our results did not confirm the hypothesis regarding HR-QOL. However, worsening psychological condition affects HR-QOL because the EQ-5D-5L includes the dimension of anxiety/depression. We considered that the increasing negative impact on mental health must decrease HR-QOL among non-permanent workers. Employment security is an important factor in maintaining the mental health of non-permanent employees. Increasing unemployment could increase suicide rates during and after the COVID-19 outbreak. We suggest that unemployment is a factor that negatively impacts mental health. A previous study indicated that long working hours are associated with an increased risk of depression in Japan. Our multiple linear regression analysis showed similar results [23].

Twenge et al. [24] reported that the prevalence of depression increased slightly in the United States from April 2020 to May 2020. We were unable to estimate the prevalence of diagnosed depression. However, half of the subjects in our data identified depressive symptoms, as indicated by CES-D scores of 16 points or above. Therefore, we considered that this is not an optimistic situation, and an increase in unemployment could lead to an increase in the incidence of depression in Japan in the near future.

Web-based surveys are reliable methods for epidemiological research [25, 26]. However, this study had several limitations. First, approximately 25% of subjects were excluded from the follow-up survey because we were unable to obtain a second response through a web survey. Younger participants were not likely to respond to the follow-up survey. Therefore, some selection bias remained in terms of follow-up data compared with initial survey data. However, we believe that such selection bias had minimal impacts on our results because we ensured adequate representation of the Japanese population in the initial survey. Second, we excluded self-employed individuals and civil servants from the analysis as this study targeted workers employed by commercial companies. Civil servants were considered to work in public service, and self-employed individuals typically worked independently. The number of subjects was limited in our follow-up data, so further data collection will be necessary to examine workers’ mental health conditions in future research.

Finally, in the follow-up questionnaire, unemployment was defined as loss of a job or discharge during the state of emergency. We were unable to collect more information regarding reasons for unemployment. Moreover, we were unable to use an additional approach to verify the respondents’ socioeconomic statuses or clinical histories because of the anonymous self-reported nature of the survey. Despite these limitations and a short-time prospective investigation, the statistical analysis presented in this study can serve as important information for future health and economic policies related to the COVID-19 crisis in Japan.

After the state of emergency was declared, the Japanese government encouraged citizens to refrain from non-essential travel and to avoid going out unless necessary. Many public and commercial facilities, excluding essential businesses, were strongly requested to close. Although the restrictions were not forceful measures like the lockdowns in some foreign countries, most Japanese people exercised self-restraint until the state of emergency was lifted. Japan succeeded in controlling the virus at the end of June. However, the number of unemployed workers due to COVID-19 was estimated to be 48,206 on August 25, according to the Ministry of Health, Labour, and Welfare in Japan [27, 28]. Japan seems to be facing a second wave of COVID-19; hence, the difficult situation might continue in the medium- to long-term period. Unemployment is expected to increase in specific industries such as manufacturing, food service, and tourism. Thus, we should carefully observe the changes in mental health and suicide rates.

## Conclusion

In conclusion, this study found that mental health of non-permanent workers was not negatively affected by the COVID-19 state of emergency in Japan. We suggest that unemployment history was a factor associated with decreasing mental health, and about 10% of non-permanent workers experienced unemployment between the two periods. The COVID-19 crisis is still in its initial phase; systematized policies, including infection control measures and also economic measures, are required to ensure that the mental health of general workers does not worsen. More studies are needed to evaluate the long-term mental health consequences and the incidence of depression during the COVID-19 crisis in Japan.

## Data Availability

The data that support the findings of this study are available from the corresponding author upon reasonable request.

## Abbreviations

OECD: Organization for Economic Co-operation and Development
DSM-5: Diagnostic and Statistical Manual of Mental Disorders – Fifth Edition
HR-QOL: Health-related quality of life
CES-D: Center for Epidemiologic Studies Depression Scale
SOC: Sense of coherence
